# Chemistry and Structure-Activity Relationship of the Styrylquinoline-Type HIV Integrase Inhibitors

**DOI:** 10.3390/molecules15053048

**Published:** 2010-04-27

**Authors:** Jean-François Mouscadet, Didier Desmaële

**Affiliations:** 1 LBPA, CNRS, Ecole Normale Supérieure de Cachan, France; E-Mail: mouscadet@lbpa.ens-cachan.fr; 2 Faculté de Pharmacie, Université Paris-Sud, UMR CNRS 8076 BIOCIS, Châtenay-Malabry, France

**Keywords:** AIDS, HIV-1, integrase, inhibitor, styrylquinoline

## Abstract

In spite of significant progress in anti-HIV-1 therapy, current antiviral chemo-therapy still suffers from deleterious side effects and emerging drug resistance. Therefore, the development of novel antiviral drugs remains a crucial issue for the fight against AIDS. HIV-1 integrase is a key enzyme in the replication cycle of the retrovirus since it catalyzes the integration of the reverse transcribed viral DNA into the chromosomal DNA. Efforts to develop anti-integrase drugs started during the early nineties, culminating with the recent approval of Raltegravir. The discovery and the development of the styrylquinoline inhibitor class was an important step in the overall process. In this review we have described the key synthetic issues and the structure-activity relationship of this family of integrase inhibitors. Crystallographic and docking studies that shed light on their mechanism of action are also examined.

## Abbreviations

HIV: Human immunodeficiency virus
IN: Integrase
INSTI: integrase strand transfer inhibitor
INBI: integrase binding inhibitor
SQL: styrylquinoline
LTR: long terminal repeat
DMAP: 4-Dimethylaminopyridine
DCC: Dicyclohexylcarbodiimide
DMF: Dimethylformamide
TFA: Trifluoroacetic acid
DFT: Density functional theory
RSV: Rous sarcoma virus
CoMFA: Comparative Molecular Field Analysis
QSAR: Quantitative structure-activity relationship
AZT: Azidothymidine.

## 1. Introduction

Despite ongoing prevention efforts the number of HIV-infected individuals is still increasing mainly in the developing world. For a long time, there have been only two HIV targets for blocking viral replication, reverse transcriptase and protease. Following an initial breakthrough with the design of the fusion inhibitor, Enfuvirtide (Fuzeon®) [1], newer generations of antiretroviral drugs have emerged to complement existing treatment regimens [2]. Small molecules capable of blocking the CCR5 chemokine receptor raised considerable hope [3], nevertheless the most promising novel class of antiretrovirals is probably the integrase inhibitors [4].

**Figure 1:**
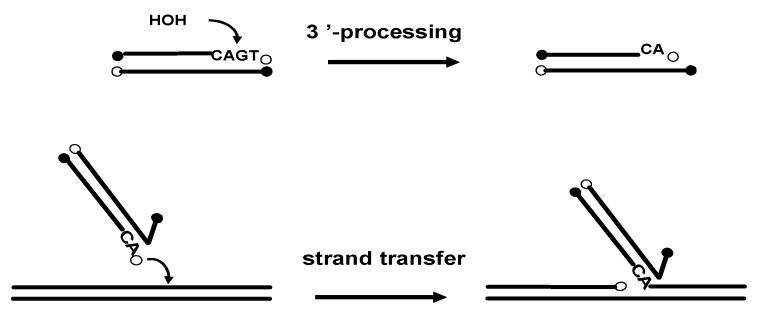
Schematic representation of the two integrase catalytic reactions (3'-processing and strand transfer).

The HIV-1 integrase (IN) is a key enzyme in the replication cycle of the retrovirus since it catalyzes the integration of the reverse transcribed viral DNA into the chromosomal DNA. Integration of the HIV-1 DNA ensures stable maintenance of the viral genome and perpetuation of the virus in the host organism, therefore making integrase an attractive target for antiviral chemotherapy. The integration process requires two distinct catalytic steps. During the first one, named 3’-processing, the integrase specifically removes two nucleotides from each 3'-end of the linear viral DNA. The second step consists in a strand transfer following the translocation of the viral DNA into the nucleus of the infected cells. During this step, IN transfers both extremities of the viral DNA into the target DNA by a one-step transesterification reaction, resulting in full-site integration (Figure 1) [5].

Since the early 90’s, many compounds that inhibit one of the two steps were identified. However, only strand transfer inhibitors (also known as INSTIs) have been shown to be potent antiviral compounds. One such compound, Raltegravir (Isentress^®^), which was developed based on early studies by Hazuda *et al*., was approved for clinical use in autumn 2007 as the first antiretroviral drug targeting the viral integrase [6]. Other compounds such as Elvitegravir or GSK1349572 and GSK1265744, are currently in advanced clinical trials [7]. Unfortunately drug-resistance variants have emerged [8]. Moreover, INSTIs differ from the other classes of antiretrovirals in that they lead to the accumulation of reverse transcribed genomes in the form of two LTRs DNA circles. Such accumulation may cause immune signals whose effects are not known [9]. INSTIs may also interfere with endogenous retroelements which replication involves an integration step, with poorly evaluated consequences [9]. Altogether, these observations have prompted not only the search for novel INSTIs but also a reassessment of the potential inhibitory activity of 3'-processing inhibitors. From this viewpoint, stryrylquinolines are a family of compounds that were found to be potent inhibitors of 3’-processing with a significant inhibitory activity against viral replication in cell cultures. In this review, we recapitulate the different steps of the development of this series and the data obtained *in vitro* and *ex vivo* that shed light on their mechanism of action.

## 2. Chemistry and Structure-Activity Relationship of Styrylquinolines *in vitro*

**Figure 2:**
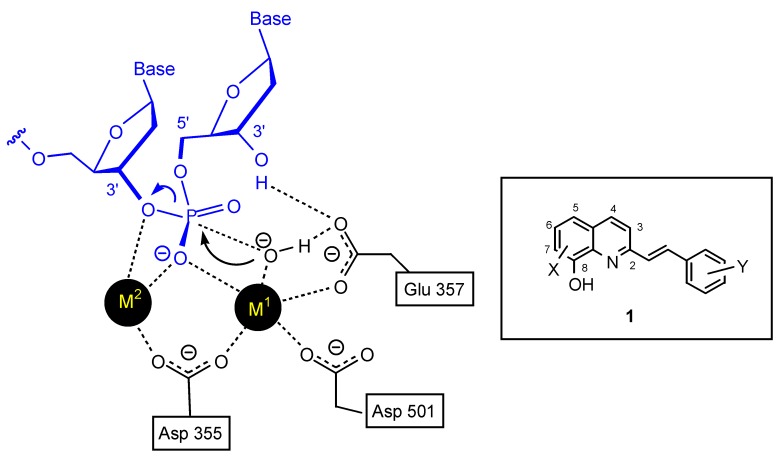
The “two-metal-ion” enzymatic mechanism for the 3'-5' exonuclease reaction of *Escherichia coli* DNA polymerase I according to the Beese and Steitz hypothesis [11] and general structure of styrylquinoline inhibitors.

3’-Processing and strand transfer reactions are both essential for the life cycle of the virus. In both cases the key step is the hydrolysis of a phosphodiester bond. However, because of the high stability of the DNA phosphodiester bond, a highly efficient catalytic process is required to provide the critical rate enhancement needed to cleave this bond [10]. Polynucleotidyl transferases, including HIV-1 IN take advantage of the catalytic effect of two-metal-ion operating in concert to achieve the tremendous rate enhancement required. For instance, in the case of the 3’-5’ exonuclease reaction of *Escherichia coli* DNA polymerase I, Beese and Steitz [11] have suggested that the phosphodiester bond cleavage is promoted by two divalent cations separated by about 3.9 Å, the first one (M^1^) initiates the formation of the hydroxide ion, whereas the second (M^2^) directly ligates the leaving group atom, thereby facilitating the bond breakage step by neutralizing the developing negative charge (Figure 2). Although the exact nature of the metal cations is unknown in the case of HIV-1 integrase the crystal structure of Mg(II)-complexed core domain of HIV-1 IN shows that one Mg^2+^ ion is coordinated by two of the three critical active site residues (Asp-64 and Asp-116) [12]. It is probable that the second ion binds only in the presence of the DNA substrate [13].

On this basis, polyhydroxylated SQLs which embedded in their structure two chelating subunits were designed to chelate divalent metal ion(s) present in the catalytic core domain of HIV-1 IN. SQLs, exemplified by **1**, (Figure 1) were found to be potent HIV-1 IN inhibitors in *in vitro* experiments. On the other hand they block the replication of HIV-1 in cell culture and are devoid of cytotoxicity [14]. Following this initial finding, a program of systematic chemical modification of the hit compounds was undertaken since receptor based design of novel HIV-1 inhibitors was hampered by the lack of complete three-dimensional bioactive structure of HIV-1 integrase. More than 150 new analogues were prepared and biologically investigated in an attempt to delineate the structure-activity relationship of this family. Modifications were initially performed on the quinoline ring since it was recognized that this moiety constituted the main pharmacophore. Further modifications and elaborate substitutions at various sites of the ancillary ring were next studied. Finally some modulations of the spacer were examined.

### 2.1. Discovery of the 8-hydroxy-quinoline-7-carboxylic acid- pharmacophore

In line with the initial proposal that a metal ion chelator might act as integrase inhibitor, 8-hydroxyquinoline derivatives were screened in both 3'-processing and strand-transfer assays. However, a complete lack of inhibitory potency was observed with the parent 8-hydroxyquinoline (**2a**) or 8-hydroxyquinaldine (**2b**) and with their *O*-alkylated and *O-*acylated derivatives. Modulation of the quinoline ring was therefore undertaken taking advantage of the 8-hydroxyl group. 

**Scheme 1:**
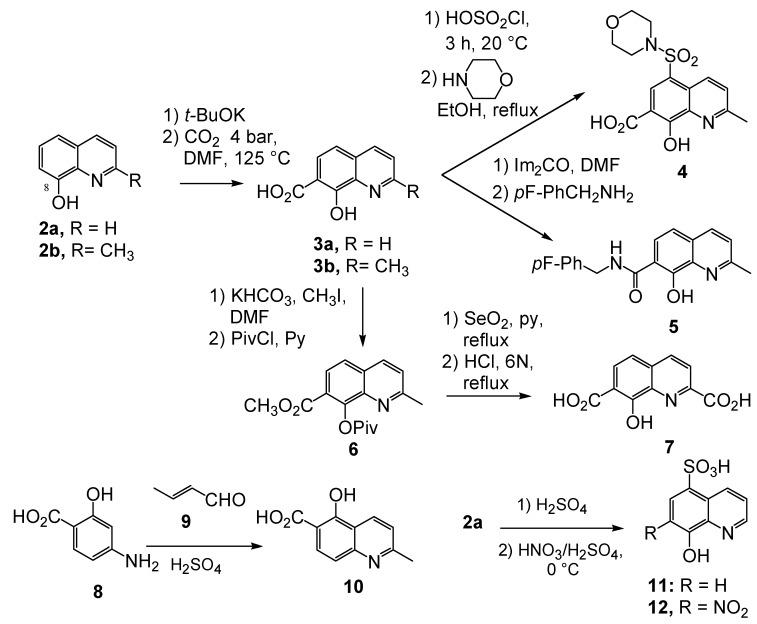
Synthesis of various quinoline and quinaldine derivatives.

Kolbe-Schmitt carbonatation according to a previously reported procedure provided acids **2a, 2b** in 20–30% yields (Scheme 1) [15]. Latter on, an improved 60% yield of **2b** was obtained according to a modified protocol in which the potassium phenoxide, formed in anhydrous condition, was treated at 125 ºC under 4 bar of carbon dioxide [16]. Functionalization of the C-2 methyl group required protection of the salicylic moiety. Thus, chemoselective methylation of the carboxylic group was achieved by alkylation using methyl iodide and potassium bicarbonate as base [17]. After protection of the C-8 phenol group as a pivalate ester, selenious anhydride oxidation in pyridine of the fully protected quinaldine **6** and acidic deprotection yielded diacid **7** [18]. Chlorosulfonylation of the C-5 position using neat chlorosulfonic acid at room temperature followed by condensation with morpholine provided the C-5 sulphonamide **4 **in 50% yield [19]. Amidification reaction of the C-7 carboxyl group was quite challenging in this series. For example, DMAP/DCC-catalyzed condensation of **3b** with 4-fluorobenzylamine delivered **5** in only 26% yield [20]. After many unsuccessful attempts, it was finally found that carboxyl activation using *N,N'*-carbonyl diimidazole followed by primary amine addition gave an improved 60% yield of quinolinecarboxamide **5** [19]. 

Looking for alternative scaffolds, Polanski *et al*. prepared the quinaldine **10** in which the salicylic moiety was shifted to the opposite side of the quinoline ring using Doebner-Miller condensation of 4-aminosalicylic acid with crotonaldehyde. Furthermore, Polanski studied the introduction of a nitro group at C-7 instead of the carboxylic acid. Direct sulfonation of 8-hydroxyquinoline (**2a**) gave **11** which was further reacted with sulfonitric acid to yield the 8-hydroxy-7-nitro-quinadine-5-sulfonic acid (**12**) (Scheme 1) [21]. 

The biological evaluation of these simple quinoline derivatives on both 3'-processing and strand transfer reactions is depicted in Table 1. Thus, 8-hydroxyquinaldine (**2b**) was found inactive. Adding a carboxylic acid group at C-7 (**3a,b**) did not improve the inhibitory potency of the quinoline scaffold. Nevertheless a promising observation was made in this series: quinaldine **4 **bearing a salicylic function at C-7, C-8 and a sulphonamide at C-5 exhibited a low but undeniable activity [19]. Replacement of the carboxyl by another strong electro-withdrawing group such as a nitro group (compound **12**) suppressed completely the activity [21]. When the salicylic system was moved from the C-7, C-8 position to the C-5, C-6 as in **10**, a discrete but significant activity was observed making this compound a promising scaffold [21]. Amide **5** derived from 8-hydroxyquinaldine (**2b**) was found completely devoid of activity. This result was in strong contrast with the Merck finding that 8-hydroxyquinoline and 7-carboxamide[1,6]naphthyridin-8-ols such as **13** and **14**, substituted at C-7 with a large lipophilic residue greatly inhibited strand-transfer [22,23]. 

Thus, despite their similarity, the 2-substituted quinoline derivatives behaved differently than the unsubstituted parent compounds. The origin of this dichotomy is not clear. As a hypothesis, it may be assumed that two different orientations of the oxine in the enzyme core coexist. In the first one, the nitrogen atom of C-2 unsubstituted quinolines such as **13** or **14** fitted in a sterically restricted area, whereas a wide lipophilic space is available for large aromatic groups around the C-7 carbon allowing additional hydrophobic interactions. A simple methyl or carboxyl group at C-2 (as in **5 **and **7**) impaired such an orientation; hence the quinoline system took an alternative orientation in which a polar group at C-7 such a carboxylic acid greatly improved the binding. These two distinct sites may also account for the different biological properties between the styrylquinoline class which behaved as integrase binding inhibitors (INBIs) and the 8-quinolines and 8-naphthyridines developed by Merck and Shionogi which were found to be highly potent strand-transfer inhibitors (INSTIs).

**Table 1:** HIV-1 IN inhibitory potency of the quinoline scaffold.

Compound		3’ProcessingIC_50_ (μM)	Strand Transfer IC_50_ (μM)	Reference
**2b**	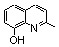	>100.000	>100.000	14
**3a**	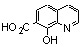	>100.000	>100.000	19
**3b**	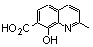	>100.000	114.000	14
**4**	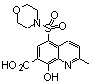	77.000	untested	19
**7**	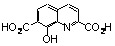	>100.000	untested	19
**12**	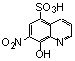	>100.000	untested	21
**10**	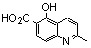	47.000	untested	21
**5**	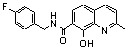	>100.000	untested	20
**13**	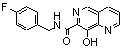		0.033	23
**14**	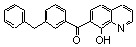		0.370	22

### 2.2.Modulation of the C-8 substituent of the styrylquinoline inhibitors

**Scheme 2:**
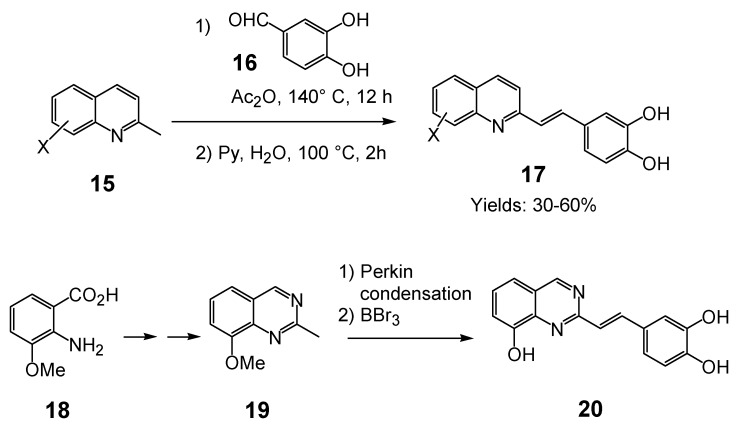
Synthesis of 8-substituted 3',4'-dihydroxystyrylquinolines.

In the quest for more potent inhibitors the introduction of an additional metal-chelating system was thus studied. An aryl nucleus possessing various hydroxy substitution patterns linked by means of an appropriate central spacer was chosen for the second chelating subunit, on the basis of the observation that the most potent HIV-integrase inhibitors known to date generally contained an ortho-dihydroxylated (catechol-type) aromatic ring. All compounds were prepared by condensing the given quinaldine with 3,4-dihydroxybenzaldehyde (**16**) according to a Perkin-type condensation as depicted in Scheme 2 [14]. Similarly, Lee *et al*. prepared the corresponding styrylquinazoline **20** in 5% overall yield by condensation of 3,4-dihydroxybenzaldehyde with the 2-methylquinazoline **19 **which is easily available from anthranilic acid (**18**) [24]. 

The influence of the C-8 substituent on the 3',4'-dihydroxystyrylquinoline scaffold is summarized in Table 2. 

**Table 2:** HIV-1 IN inhibitory potency of various 3',4'-dihydroxystyrylquinolines.

Compd.Nb	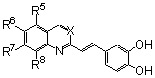					3’-Processing IC_50_ (μM)	References
	R^5^	R^6^	R^7^	R^8^	X		
**21**	H	H	H	H	CH	>100.0	14
**22**	H	H	H	OH	CH	7.4	14
**20**	H	H	H	OH	N	57.0	24
**23**	H	H	H	OAc	CH	>100.0	14
**24**	H	H	H	NO_2_	CH	>100.0	14
**25**	H	H	H	NH_2_	CH	>100.0	14
**26**	H	H	H	CO_2_H	CH	>100.0	25
**27**	CO_2_H	H	H	H	CH	>100.0	25
**28**	H	CO_2_H	H	H	CH	>100.0	25
**29**	H	H	CO_2_H	H	CH	>100.0	25
**30**	CO_2_H	H	CO_2_H	H	CH	>100.0	25
**31**	OH	CO_2_H	H	H	CH	>100.0	19
**32**	CO_2_H	H	H	OH	CH	7.0	19

Although the parent compound **21** is devoid of activity, the 8-hydroxystyrylquinoline **19** displayed a modest but encouraging *in vitro* activity. However, the replacement of the free hydroxyl group by an acetoxy substituent completely abolished the activity (compare **22** with **23**) [14]. Hence, the inhibitory potency is clearly associated with the presence of “free” phenolic hydroxyl group at C-8 since a nitro or a carboxyl groups at C-8 were unable to assure integrase inhibition. SQLs **23-31** proved to be completely devoid of activity, despite the presence of a catechol moiety in all these compounds [14,25]. Unexpectedly, no noticeable integrase inhibition was observed when an amino group was fixed at C-8, indicating that a simple hydrogen bond donor residue is inefficient in promoting inhibition. The influence of the carboxylic residue in the absence of the 8-hydroxy group was also briefly investigated [25]. Acids **26-29** were found to be completely devoid of integrase inhibition potency, a result indicating that IN affinity seems to be strongly connected with the Mg^2+^ complexing ability of the oxine moiety. The influence of additional polar group such as carboxyl group at C-5 of the 8-hydroxyquinoline ring as in **32 **might be correlated with an increase acidity of the C-8 phenol group which led to higher ion-chelating potency. Finally, modest inhibitory potency was found for the styrylquinazoline (**20**) compared to SQLs, unfortunately neither the influence of an additional carboxyl group at C-7 nor the antiviral evaluation were reported [24]. 

### 2.3.Modulation of the C-7 substituent on the 8,3',4'-trihydroxystyrylquinoline scaffold

The introduction of a polar group at C-7 was the key breakthrough in the styryquinoline class design story. Thus, when an additional nitrile group was introduced at the C-7 position in the quinoline half (compound **34**, Table 3) micromolar levels of anti-integrase inhibition were reached. The starting material in the preparation of nitrile **34** was the known 7-carbaldehyde-8-hydroxy-2-methylquinoline which was first converted into oxime **33**. Concomitant dehydration of the oxime function of **33 **took place during the Perkin condensation with 3,4-dihydroxybenzaldehyde (**16**), affording carbonitrile **34** in 71% yield. Similarly, styrylquinoline **35** resulted from the condensation between quinaldine **3b** and aldehyde **16**, followed by hydrolysis of the crude product by using a pyridine-water mixture [14]. 

The introduction of a carboxylic acid group at C-7 (compound **35**) produced potent HIV-1 IN inhibitors. Furthermore, beside this good *in vitro* potency, this compound gave rise to a significant antiviral effect as measured by β-gal assay, whereas no toxicity on either infected or non-infected cells was observed. In sharp contrast, compound **54** in which the 7-carboxyl group was replaced by a 7-carbomethoxy group, was completely devoid of biological activity [26]. The latter result clearly indicated that a free carboxylic group greatly improved integrase inhibition, suggesting that a Lewis base containing group at C-7 is need for good inhibition potency. Interestingly enough, an amide function is unable to play such a role, since styrylquinoline carboxamides **55 **(Table 3), obtained through direct condensation of the requisite amine with **35**,exhibited no noticeable HIV-1 IN inhibition when tested up to 300 μM [19]. The significant loss of HIV-1 IN inhibitory activities of these compounds compared to **35** indicated that the increased size of such compounds seems not suitable to fit into the catalytic pocket of the active site although these compounds possess two pharmacophores that can bind with Mg^2+^. The importance of the basic nitrogen in the styrylquinoline scaffold has been illustrated by comparison with the corresponding styrylbenzofuran **37** which was found to be 20-fold less potent that **35**. The latter was prepared with 20% yield through a Wittig type olefination using phosphonium **36** and 3,4-dihydroxybenzaldehyde (**16**) [27].

**Scheme 3:**
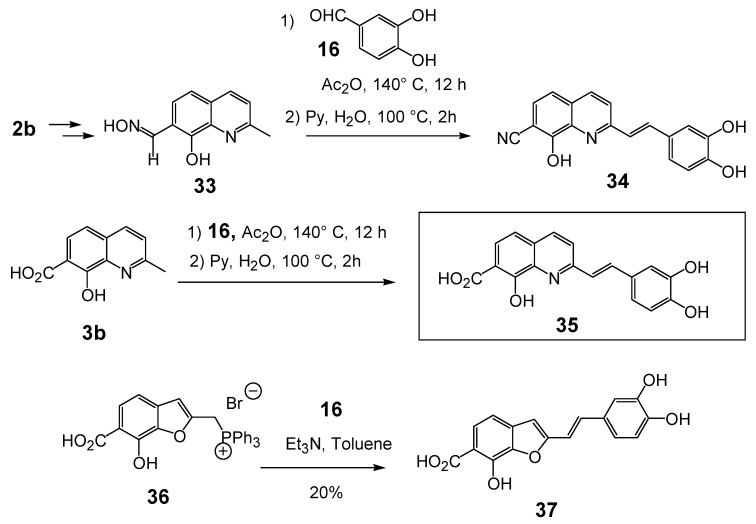
Synthesis of 8-hydroxy-3',4'-dihydroxystyrylquinoline 7-carbonitrile ** (34**), 7-carboxylic acid **35** and benzofuran **37**.

In line with the above results, well known isosters of carboxylic acid such as phosphonic acid and tetrazole group were introduced at the C-7 position of the styrylquinoline scaffold to evaluate their influence (compounds **43** and **46**). Furthermore, the distance between the quinoline ring and the acid function introduced at C-7 was modulated by introducing an ethylenic spacer as in **42**.

**Scheme 4:**
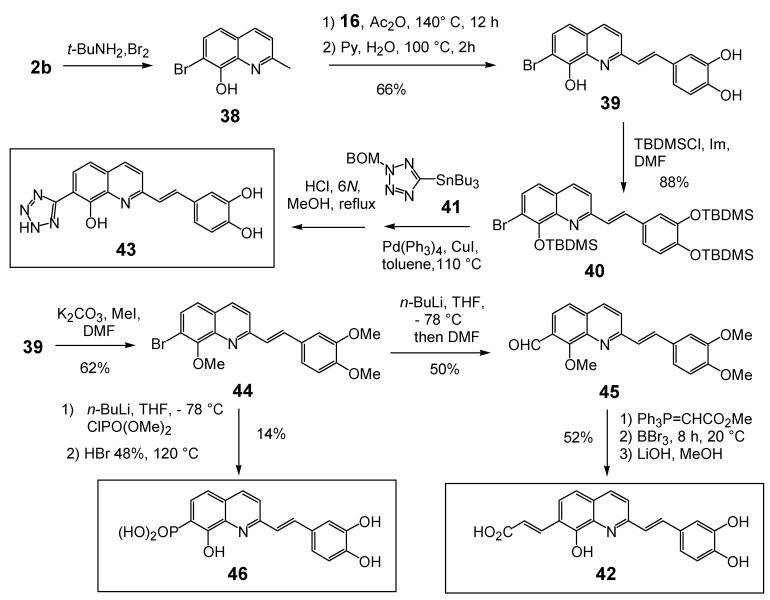
Synthesis of C7-modified styrylquinolines **42, 43 **and **46**.

The syntheses of **43** and **46 **are depicted in Scheme 4. First, the crucial regioselective bromination of the C-7 position of 8-hydroxyquinaldine **2b** was carried out using the *tert*-BuNH_2_·Br_2_ complex [28]. Condensation of 7-bromo-8-hydroxyquinaldine (**38**) with 3,4-dihydroxybenzaldehyde (**16**) and protection of the hydroxyl groups gave SQLs **40** and **44** respectively. Stille cross-coupling reaction of bromide **40** with the stannyl tetrazole subunit **41 [29]** followed by fluoride deprotection of the silyl groups provided the desired 7-tetrazole-styrylquinoline **43**, albeit in modest yield. Similarly the fully protected styrylquinoline **44** was metallated with *n*-butyllithium and treated with DMF. The resulting aldehyde **45** was condensed with carbomethoxytriphenylmethylenephosphorane and deprotected with BBr_3_ to provide the unsaturated acid **42** [30]. In turn, phosphonic acid **46** was prepared in 15% overall yield from **44** through sequential lithium bromine exchange with *n*-butyllithium and treatment of the resulting lithio derivative with diethylchlorophosphate, followed with HBr deprotection [30].

Unexpectedly, the tetrazole **43** did not show any improved integrase inhibition activity whereas the phosphonic acid **46** was completely inactive. On the other hand, the α,β-unsaturated acid **42** was found equipotent than the parent compound **35**, suggesting that the geometric constraints for the additional interaction at the C-7 carbon center were not stringent. 

Structurally, compound **47** only differs from lead inhibitor **35** in the presence of an additional keto group at C-7. The factor that has stimulated interest in synthesizing styrylquinoline **47** has been the striking structural analogy between the HOOC-CO-C=C(OH)- system and the enol form of the α,γ-diketo acid pharmacophore found in the potent Merck inhibitor L 731,988 [31]. 

**Scheme 5:**
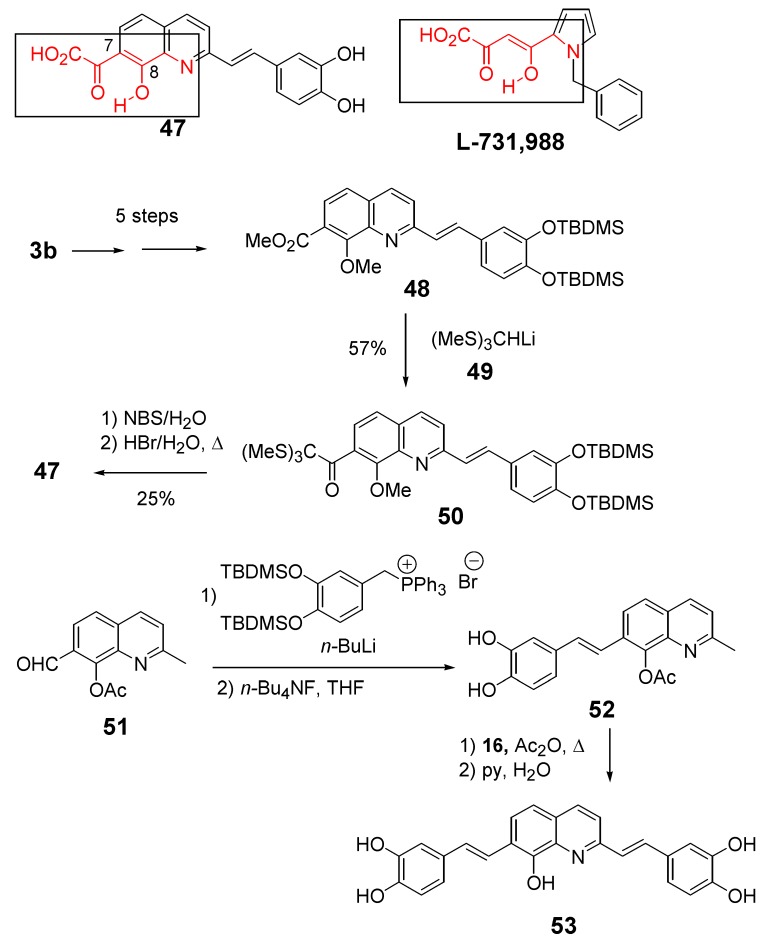
Synthesis of 7-ketoacid **47 **and bis-catechol **53**.

The introduction of this critical moiety has taken advantage of the one-carbon elongation of esters using the anion of tris(methylthio)methane **49 **according to the Scheme 5. In contrast with parent compound **35**, this keto acid exhibited a complete lack of *in vitro* inhibitory potency (IC_50 _>100 μM) [32]. This result confirmed that structural requirements of INBIs strongly differ from those of diketoacid strand transfer inhibitors. The influence of non-acidic functional groups at the C-7 position of the quinoline half was also studied. Thus, 7-(2-furyl)styrylquinoline **56** available in 24% overall yield by Stille coupling of bromide **38** with 2-(tributylstannyl)furan followed by Perkin-type condensation, was found inactive [30]. This result is in line with the hypothesis of the requirement of a Lewis base atom at the C-7 center. On the other hand the quinoline derivative **53** bearing two similar dihydroxyphenylethenyl subunits at C-2 and C-7 was found equally potent that the lead compound **35** [14], indicating that the catechol group might play the same role that the C-7, C-8 hydroxy acid group and that the exact orientation of the molecule in the active site is still in debate (Table 3).

**Table 3:** HIV-1 IN inhibitory potency of various 7-substituted 8,3’,4’-trihydroxystyrylquinolines.

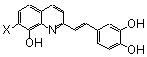		3’-Processing IC_50_ (μM)	Strand Transfer IC_50_ (μM)	Reference
**34 **	X = CN	3.0	Untested	14
**35**	X = CO_2_H	2.4	1	14
**54**	X = CO_2_CH_3_	>100	>100	26
**42**	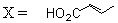	2^a^		30
**43**	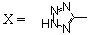	Untested	2.4	30
**46**	X = (HO)_2_PO	Untested	>100	30
**47**	X = HO_2_CCO	100	>100	32
**53**	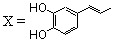	2.3	Untested	14
**56**	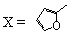	100	Untested	30
**55**	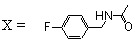	>300	Untested	19

^a^ overall binding assay.

**Scheme 6:**
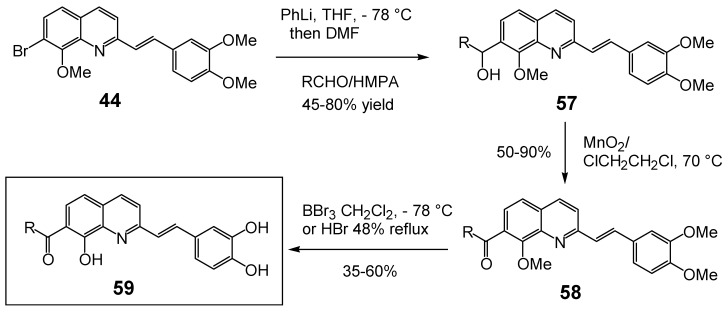
Synthesis of 7-aroyl-styrylquinolines **59**.

Since 7-aroyl-8-hydroxyquinoline derivatives such as **14 **(Table 1) were found to be highly efficient strand transfer inhibitors by Merck [22], a series of 7-acyl/aroyl SQLs were prepared and evaluated. All these compounds were obtained in 10–40% overall yield from the fully protected 7-bromo-styrylquinoline **44** through sequential lithium/halogen exchange (PhLi, Et_2_O, -78 ºC), condensation of the resulting 7-lithio derivative with the given aldehyde, MnO_2_ oxidation and finally demethylation using either HBr at reflux or BBr_3_ at -78 ºC (Scheme 6) [33].

In sharp contrast with SQLs of type **35** displaying a carboxylic acid group at C-7, most of these compounds proved to be inactive in 3'-processing or overall binding assays as shown in Table 4 probably due to the steric bulk of the C-7 substituent. In line with this hypothesis, the acetyl substituted compound **59d **constituted a notable exception. However, this result could not directly be compared since the ancillary half is modified [34]. The acid substituted derivatives **59n-p** constituted another exception, reinforcing the previous finding that carboxylic acid residues improved the anti-IN activity [33]. 

**Table 4:** *In vitro* integrase inhibition activities of the 7-aroyl-styrylquinolines derivatives.

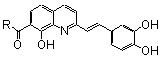	3’-Processing IC_50_ (μM)	Strand Transfer IC_50_ (μM)	References
**59a**, R = H	82.0	Untested	30
**59b**, R = Ph	50.0^a^	Untested	33
**59c**, R = 1-Naphthyl	92.0	Untested	33
**59d^a^**, R = CH_3_	0.2	0.2	34
**59e**, R = C_8_H_17_	>100.0 ^b^	Untested	33
**59f**, R = PhCH_2_CH_2_	>100.0 ^a^	Untested	33
**59g**, R = 4-NO_2_Ph	100.0	>100.0	33
**59h**, R = 2-NO_2_Ph	>100.0	70.0	33
**59i**, R = 3,4-F_2_Ph-	24.0	>100.0	33
**59j**, R = 4-pyridyl	>100.0 ^a^	Untested	33
**59k**, R = 3-pyridyl	>100.0	>100.0	33
**59l**, R = 2*-*OH-Ph	>100.0	>100.0	33
**5m**, R = 4*-*OH-Ph	65.0	>100.0	33
**59n**, R = 4-(HO_2_C)Ph	0.2	Untested	33
**59o**, R = 3-(HO_2_C)Ph	0.2	Untested	33
**59p**, R = 2-(HO_2_C)Ph	0.3	Untested	33

^a^ 1,3,4-trihydroxyphenyl instead of the 3,4-dihydroxyphenyl ancillary ring. ^b^ Overall binding assay.

### 2.4. Functionalization of the C-5 quinoline position

Given the positive influence of a polar group at the C-7 position of the quinoline ring on the anti-integrase potency the introduction of an additional group at the C-5 position was studied. 5,7-Dihalostyrylquinolines **60** and **61** (Table 5) were easily prepared through Perkin condensation of the commercially available 5,7-dihalo-8-hydroxyquinaldines with aldehyde **16** [26]. New structural variants possessing an additional carboxyl group at the C-5 carbon atom, either directly bound to the quinoline ring or through an ethylenic spacer were also prepared. The synthesis of 5,7-quinaldinedicarboxylic acid (**69**) took at profit the palladium catalyzed alkoxycarbonylation reaction of 7-iodo-quinaldine **67** which is available in two steps from **66**. Perkin-type condensation of **69** with 3,4-dihydroxy-5-methoxybenzaldehyde and hydrolysis gave the diacid **63** in 21% overall yield. Heck reaction of bromoquinaldine **70** with methyl acrylate afforded unsaturated ester **71 **which was next engaged into Perkin condensation with **16** and hydrolysis to provide the 5-propenoic acid derivative **64** in 32% overall yield [34]. 

**Scheme 7:**
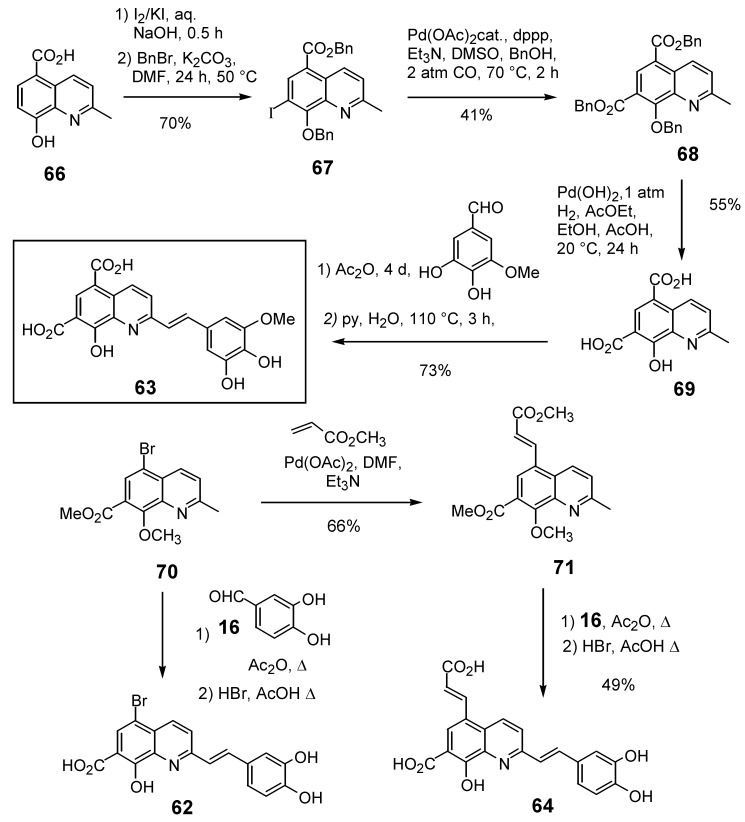
Synthesis of C-5 functionalized styrylquinolines** 62** and **64**.

**Scheme 8:**
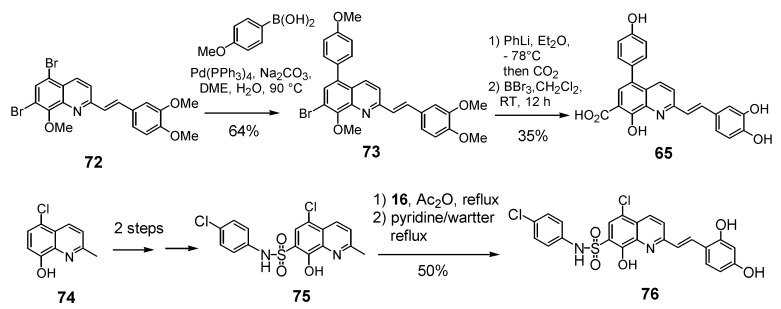
Synthesis of 5-arylstyrylquinoline **65 **and 7-sulfonamide **76**.

The 7-arylstyryquinoline **65** was obtained in 22% overall yield using Suzuki cross-coupling of 4-methoxyboronic acid with 5,7-dibromostyrylquinoline **72**. The remarkable regioselectivity may have its origin in the low reactivity of the C-7 bromine atom in the oxidative addition step due to the presence of a vicinal methoxy group. Lithium bromine exchange reaction, followed by carbon dioxide condensation with the resulting 7-lithio derivative afforded after HBr deprotection the 5-aryl-styrylquinoline **65** (Scheme 8) [19]. Assuming that a sulfonamide group may engage a H-bonding network as a carboxylic acid, Zen *et al.* synthetized a series of 2-styrylquinoline-7-sulfonamides exemplified by **76**, starting from the 7-chloro-8-hydroxyquinaldine (**74**), according to a four-step sequence involving chlorosulfonation, amination, Perkin-type condensation with 3,4-dihydroxybenzaldehyde (**16**) and hydrolysis [36].

Not surprisingly, the dichloro and dibromoquinoline derivatives **60** and **61** were found to be either inactive or poorly active. On the other hand, bromoacid **62**, which displays a carboxyl group a C-7, showed roughly the same activity that the unsubstitued C-5 derivative **35**. The introduction of a carboxyl residue at C-5 either directly (compound **63**) or through an ethylenic spacer (compound **64**) was highly beneficial to the *in vitro* activity, since diacid **63** was one of the most potent styrylquinoline prepared so far [34]. On the other hand, sulfonamide **76** prepared by the Zen’s group, turned out to be totally inactive. Clearly the bulky sulfonate group, although it might provide a hydrogen-bond is unable to play the same role that the 7-carboxyl group which is probably ionized at the pH of the integrase assay (Table 5) [36].

**Table 5:** HIV-1 IN inhibitory potency of 5,7-disubstituted -8-hydroxystyrylquinoline derivatives.

Compd. Numb.	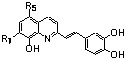		*In vitro* biological activity		Reference
	R^5^	R^7^	3’-Processing IC_50_ (μM)	Strand TransferIC_50_ (μM)	
**60**	Cl	Cl	>100.00	>100.00	26
**61**	Br	Br	21.00	33.00	19
**62**	Br	CO_2_H	5.00	Untested	30
**63^a^**	CO_2_H	CO_2_H	0.20	0.07	34
**64**	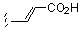	CO_2_H	1.40	1.00	34
**65**	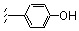	CO_2_H	>100.00^c^	Untested	19
**76^b^**	Cl	SO_2_NH*p*ClPh	>100.00	Untested	36

^a^ 3,4-dihydroxy-5-methoxyphenyl instead of the 3,4-dihydroxyphenyl ancillary ring ^b^ 2,4-dihydroxyphenyl instead of the 3,4-dihydroxyphenyl ancillary ring; ^c^ Overall binding assay.

### 2.5. Modulation of the ancillary ring

Following the initial finding that a (3,4-dihydroxyphenyl)ethenyl substituent bound to the C-2 position of the 8-hydroxyquinoline scaffold produced good integrase inhibitor a systematic program of modulation of the ancillary ring was engaged. All compounds were prepared by Perkin-type condensations of the given aldehyde with 8-hydroxy-2-methyl-quinoline-7-carboxylic acid (**3b**) according to Scheme 2. More than thirty analogues were synthesized and evaluated against integrase. 

The results obtained with compounds bearing simple phenyl groups, heteroaromatic and monosubstituted aromatic ancillary groups are depicted in Table 6. Whatever the nature of the ancillary ring, SQLs **77a-i** displayed good level of IN inhibition potency. This result seemed indicate that this part of the molecule interacts at least in part, with IN through hydrophobic or pi-type interactions. However, the chlorobenzamide group in **77k,** and at a lesser extent the thiomethyl group in **77j **were too bulky to fit in the hydrophobic pocket of the enzyme, making these compounds inactive.

**Table 6:** Influence of the ancillary ring on the HIV-1 IN inhibitory potency of 8-hydroxy-2-styryl-quinoline-7-carboxylic acids.

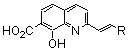		*In vitro* Biological activity		Reference
Compd. nb.	R	3’-Processing IC_50_(μM)	Strand Transfer IC_50_ (μM)	
**77a**	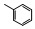	5.3	2.1	14
**77b**	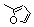	1.9	5.1	26
**77c**	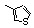	3.4	3.0	26
**77d**	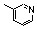	4.0	11.0	26
**77e**	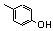	1.6	untested	26
**77f**	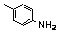	2.2	3.5	26
**77g**	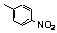	1.2	1.7	26
**77h**	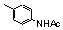	3.4	31.0	26
**77i**	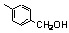	5.0	untested	25
**77j**	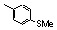	39.0	30.0	30
**77k**	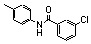	>100.0	untested	30

Results obtained with SQLs di- and trisubstituted on the aromatic ancillary ring are listed in Table 7. Beside the 3,4-dihydroxy pattern, good IN inhibitory activities were also observed with isomers, **77l-n**, with a special mention for compound **77n** in which the phenyl nucleus is 2,3-dihydroxylated. Replacement of the hydroxyl groups with fluorine atoms reduced the activity significantly but did not suppress it totally, in line with our assumption that hydrophobic interaction is the main factor *for in vitro* integrase inhibition. 

**Table 7:** Influence of the ancillary ring on the HIV-1 IN inhibitory potency of 8-hydroxy-2-styryl-quinoline-7-carboxylic acids.

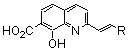		*In vitro* Biological activity		Reference
Compd. nb.	R	3’-Processing IC_50_(μM)	Strand Transfer IC_50_ (μM)	
**35**	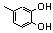	2.40	1.00	14
**77l**	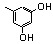	3.20	3.20	26
**77m**	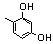	3.70	2.80	14
**77n**	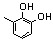	0.60	0.03	26
**77o**	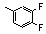	10.00	30.00	25
**77p**	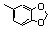	5.00	untested	25
**77q**	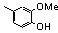	2.80	3.70	26
**77r**	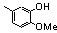	1.30	3.00	26
**77s**	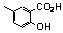	2.70	0.60	14
**77t**	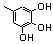	0.30	0.40	14
**77u**	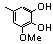	0.70	2.80	26
**77v**	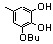	5.40	untested	26
**77w**	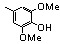	4.90	4.50	26
**77x**	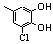	0.30	0.30	37
**77y**	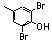	1.20	1.00	26
**77z**	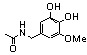	2.00	2.00	19
**77aa**	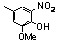	7.00	4.00	19
**77ab**	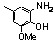	7.00	7.00	19
**77ac**	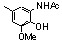	10.00	untested	19

^a^ Overall binding assay.

It is noteworthy that compared with the catechol-containing compound **35**, the same level of activity was gained with substrates **77q**,**r**,**s** in which one of the hydroxyl substituents was replaced by a methoxy or carboxyl group [14,26]. Thus, the latter examples have clearly demonstrated that the design of potent SQLs inhibitors does not necessarily require the presence of an ortho-dihydroxylated (catechol-type) aromatic moiety. An advantageous result, considering the potential stability problems associated with the pharmacological development of catechol-containing drugs.

The 3’,4’,5’-trihydroxyphenyl styrylquinoline **77t** was found highly active (3'-processing : 0.3 μM). This confirmed the previously observed trend that groups able to give hydrogen bonding improved the biological activity. Replacement of one hydroxyl by a methoxy group (compound **77u**) reduced only slightly the activity. By contrast a large lipophilic chain as in **77v** had a more severe effect. The replacement of two of thee hydroxyl groups with bromine atoms (**77y**) did not reduced drastically the inhibition potency. This result seemed indicate that an increased acidy of the phenol group is beneficial for the IN inhibition [14,26]. A hypothesis reinforced by the good inhibition potency of the chloro derivative **77x** [37]. Nitrogen containing derivatives **77aa**, **ab**, **ac** were found slightly less active.

The screening of a large number of derivatives, have thus demonstrated that the influence of the substitution on the ancillary ring is only moderate. Although hydroxyl groups had clearly a favourable effect, other hydrogen bond donating groups can be equally effective. Thus the choice of a lead compound for further development in the styrylquinoline series will be more surely dictated by the antiviral properties and physico-chemical behaviour than based on the structure-activity relationship of the ancillary ring.

### 2.6. Modulation of the spacer

**Figure 3:**
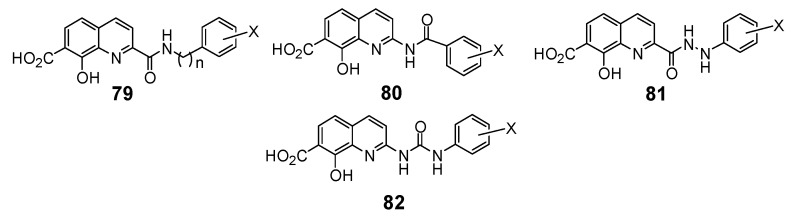
Structure of inhibitors exhibiting a nitrogen containing spacer.

In a search for new structural features able to improve the biological profile of this class of inhibitors, the linker between the two aromatic units was modified. First of all the double bond was reduced to increase the conformational liberty between the two aromatic subunits. In an attempt to optimize the binding within the active site of the enzyme, polar functions able to interact through hydrogen bond with near amino acids were intercalated between the two rings, instead of the initial olefinic linker. Thus, compounds **79–82** possessing an amide, a hydrazide or a urea spacer were prepared, keeping constant the 7-CO_2_H, 8-OH pattern on the quinoline ring (Figure 3) [18]. Synthetically, the reduced quinoline **78** was easily available through catalytic hydrogenation of the corresponding SQL **35** [26]. The others derivatives were prepared using the pivotal acid **84** available in 5 steps from 8-hydroxy-2-methyl-quinoline-7-carboxylic acid (**3b**). Activation of carboxylic group of **84** using N-hydroxysuccinimide, followed by condensation with the requisite amines **87** provided amides **79** in 15–90% overall yield, after TFA deprotection using guaiacol as *tert-*butyl cation scavenger.

**Scheme 9:**
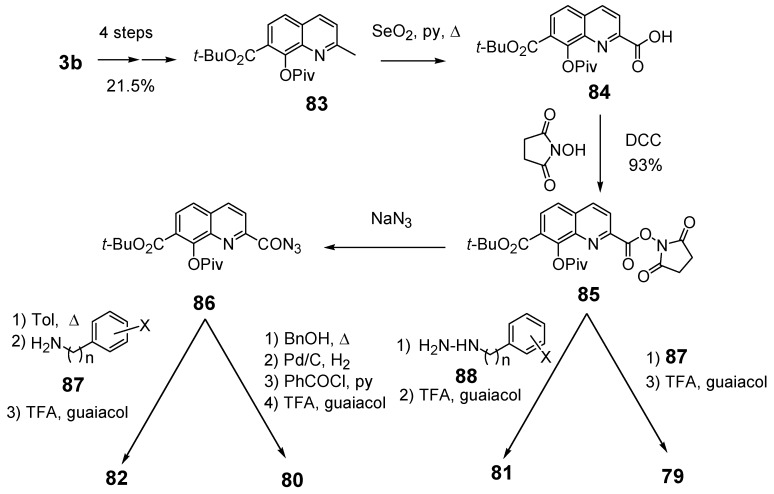
Synthetic scheme of amides **79**, **80**, hydrazide **81** and urea **82**.

Similarly condensation of **85** with hydrazines **88 **afforded hydrazides **81 **in 55–65% overall yield. On the other hand, Curtius rearrangement of acyl azide **86 **and condensation of the intermediate isocyanate with aromatic amines gave the expected urea **82** which were deprotected as previously upon treatment with TFA. Curtius rearrangement in presence of benzyl alcohol, followed by hydrogenolysis of the resulting benzyl carbamate gave the C-2 amino-derivative which was converted to retro-amide **80 **by treatment with benzoyl chloride and TFA deprotection (Scheme 9) [18].

The results of the biological evaluation of these linker-modified compounds keeping constant the 3’,4’-dihydroxyphenyl subunit are depicted in Table 8. The reduction of the central double bond did not affect the integrase inhibition potency (compound **78**). In first place, this result demonstrated that the electron circulation from the catechol system towards the quinoline nitrogen, according to a "pull-push effect" was not required for inhibition. Furthermore an increased conformational liberty appeared not to be detrimental to the inhibition potency. Quinoline-2-carboxamide **92**, which possessed a two-carbon spacer displayed a slightly improved activity compared to the corresponding styrylquinoline **35**, whereas higher the length of the arm lower the activity. In this respect, both ureas **94** and **95** were found inactive (Table 8).

**Table 8:** HIV-1 IN inhibitory potency of spacer modified inhibitors.

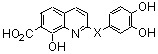		*In vitro* biological activity		Reference
Compd nb.	X	3’-Processing IC_50_(μM)	Strand Transfer IC_50_ (μM)	
**35**	HC=CH	2.4	1.0	14
**78**	H_2_CCH_2_	2.3	1.5	26
**92**	CONH	0.9	untested	18
**93**	CONHCH_2_	5.0		18
**94**	HNCONHCH_2_	>100.0		18
**95**	HNCONH	>100.0		18

To further study the influence of the ancillary ring on spacer-modified compounds, quinolines **96–106** were prepared according to Scheme 9 using various amine and hydrazine derivatives [18]. Dimeric hydrazide **106** was prepared upon treatment of *N*-hydroxysuccinimide **85** with hydrazine followed by TFA deprotection [19]. The biological evaluation of these new compounds is shown in Table 9. However in no case, were compounds with nitrogen modified spacers found to be more active than the corresponding styrylquinolines. Interestingly enough, the 3,5-dinitrophenylhydrazone derivative **104** was found to me unexpectedly active, although devoid of a hydroxyl group on the right hand. This result confirmed that the catechol moiety is not required for *in vitro* IN inhibition.

## 3. Physico-Chemical Rational of the Styrylquinoline-Integrase Interaction

### 3.1. Magnesium binding ability of styrylquinoline integrase inhibitors

Integrase is a 32-kDa protein that consists of three functional domains. The three domains are necessary for all IN activities except the disintegration, an apparent reverse reaction of the strand transfer [38], for which the catalytic core alone or truncated IN are catalytically competent. The core domain and the C-terminal domain display a DNA-binding activity and are therefore potential targets for SQLs. However, early studies have shown that the core domain-mediated disintegration reaction was efficiently inhibited by SQL compounds, with IC50 values similar to those characterizing the 3’-processing reaction, demonstrating that the SQL binding site is within the central domain of IN [14]. 

**Table 9:** HIV-1 IN inhibitory potency of spacer modified inhibitors.

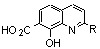		*In vitro* biological activity	
Compd. Nb.	R	3’-Processing, IC_50_ (μM)	3’-Processing activity of the corresponding SQL IC_50_ (μM)
**96**	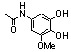	6.5	0.7 (**77u**)
**97**	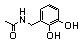	5.0	0.6 (**77n**)
**98**	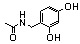	5.0	3.7 (**77m**)
**99**	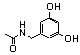	>100.0	3.2 (**77l**)
**100**	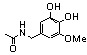	6.5	0.7 (**77u**)
**101**	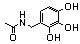	1.5	_
**102**	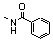	>100.0	5.3 (**77a**)
**103**	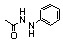	>100.0	5.3 (**77a**)
**104**	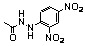	3.0	_
**105**	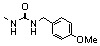	>100.0	_
**106**	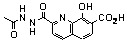	2.9	_

**Figure 4:**
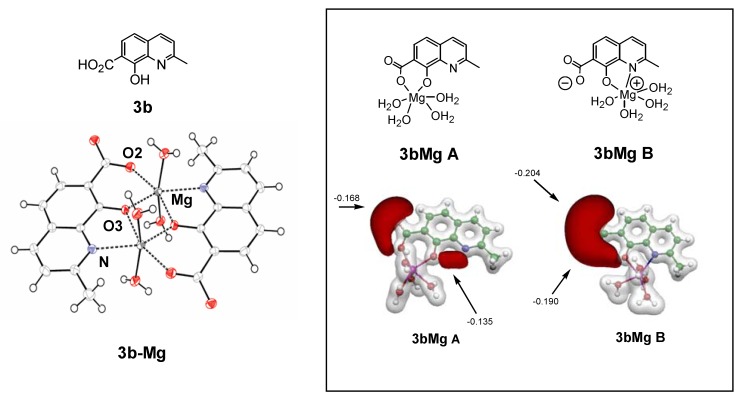
Structures of **3b** and X-ray structures of magnesium complex **3b-Mg**. DFT electrostatic potential features of putative magnesium complexes **3b-Mg A**, **3b-Mg B. **Grey and black isosurfaces correspond to +0.20 and -0.10 a.u., respectively. The arrows indicate the local minimum values of the electrostatic potential.

As hypothesized for the early development of SQLs, one possible mechanism of action is that they block the polynucleotide binding and catalytic site of HIV-1 IN through functional sequestration of the Mg^2+^ cofactor by either by the oxine moiety or by their salicylic acid part [26]. With the aim to reinforce this hypothesis, the complexing ability of SQLs for magnesium ions was verified through further experiments including the elaboration and crystallographic characterization of SQL-divalent metal complexes [39]. Unfortunately, attempts to solve the crystal structure of a variety of Mg^2+^ complexes of styrylquinolines were unsuccessful. Therefore, complexation of **3b** (Figure 4) by Mg^2+^ ion was thus investigated, bearing in mind that previous structure-activity relationship studies [14,26]. and docking procedures [40,41,42]. have identified the salicylic acid moiety of the quinoline half of SQLs as a critical pharmacophore for antiviral activity. The molecular structure of dimeric complex **3b-Mg** is depicted in Figure 4. Two tridentate ligands coordinate two Mg^2+^ cations via the carboxylato O2-atom, the phenoxo O3-atom and the quinoline nitrogen atom. N and O2 atoms are linked to one Mg^2+^ ion whereas O3 is bonded to the two metal atoms. In order to quantify and further explore the metal-chelating property of **3b** a DFT computation of the electronic structure of the complex **3b-Mg** was performed [39,43]. The most stable configuration was found for monometallic complex **3b-Mg A** where the magnesium cation is linked to both carboxyl and hydroxyl oxygen atoms. Comparatively, the complex **3b-Mg B** involving O3 and N atoms, exhibited the smallest binding energy of the Mg cation.

Although the exact mechanism by which SQL analogues exert their antiviral activity is still a matter of controversy, several studies have showed that such compounds inhibit IN at its interface with viral DNA and divalent metal(s), thereby preventing DNA-IN binding [44]. The ability for progenitor **3b** to coordinate magnesium(II) ions is consistent with such a model. We have previously demonstrated through X-ray studies that both drug **77u** and its progenitor **3b** crystallize in zwitterionic state [43]. Indeed, the carboxylate group of these molecules was found to be ionized in the solid state. This phenomenon, highlighted by the large negative electrostatic potential isosurface surrounding the carboxylate, clearly reinforces the chelating ability of these ligands towards metal cations.

### 3.2. Comparative molecular field analysis and docking studies

**Figure 5:**
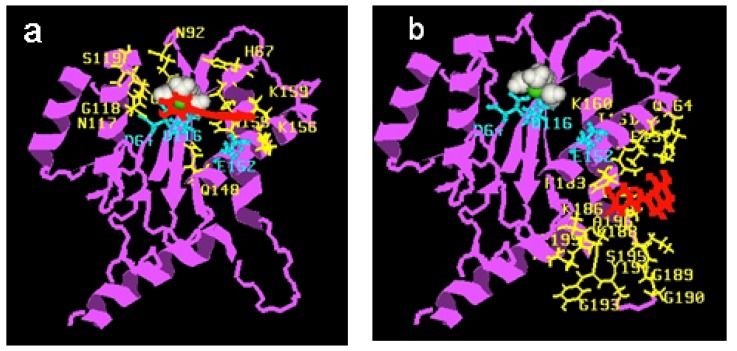
View of one SQ-IN complex typical conformation belonging to each cluster: a, cluster 1; b, cluster 2. The protein is shown in magenta, Mg^2+^ in green, water in white; drug in red, the catalytic triad (Asp64-Asp116-Glu152) in cyan (sticks and balls) and contacting residues are in yellow.

Initial modelling studies were conducted using the Rous sarcoma virus (RSV) integrase core domain [40,41]. *Ab initio* studies were first performed in order to elucidate the conformation and charge distribution of **77q** (Table 7). The *s*-*trans* conformation as usually represented in the schemes was found the most stable. The same conformation was observed in the crystal structure [43]. However, the *s-cis* confirmation was found to be higher in energy by only 0.3 kcal/mol. Thus, we can assume that considerable flexibility around this bond exists in biological medium. Docking of the styrylquinoline **77q** shows that the inhibitor binds closely to the Mg^2+^ cation making contacts with the D64, N122, N149, Q153, N160 residues [40,41]. In a more recent work [44], compound **35** (Scheme 3) was docked on the HIV-1 IN catalytic core. After rigid docking and minimization, the best target-drug complexes were selected according to their interaction energies (coulombic and Van der Waals). Three main cluster sites were identified. The contacting residues of cluster 1 overlap the catalytic triad (Figure 5**a**). Cluster 2 is actually made of a succession of overlapping sites and is spread over a large region of the protein surface (Figure 5**b**) whereas a little number of conformations forms the cluster 3 (not shown). As expected, the negatively charged drug bound to lysine-rich regions. Residues K156 and K159 Q148 and S119 in cluster 1 have been previously found to be involved in DNA binding [45,46,47]. In contrast, no residues from cluster 2 are known to be essential for DNA binding. In conclusion, from computational simulations, it seems that cluster 1 is the primary binding site for SQLs as already observed with the RSV core domain. This cluster, which corresponds to one of the two proposed DNA-binding sites, contains the catalytic triad and several key residues for DNA-binding [48]. 

Using a fluorescence anisotropy-based DNA-binding assay it was confirmed that SQLs prevent recognition of viral by IN, demonstrating that the 3’-processing inhibitory effect is in fact due to the ability to impair DNA binding by competitive inhibition. This mechanism strongly differs from the one of INSTIs which do not bind efficiently to the active site of the free IN but display a strong affinity for the IN/viral DNA complex [31,49].

Furthermore, attempts were made to obtain a Comparative Molecular Field Analysis (CoMFA) model to predict the activity and the rational binding mode of styrylquinoline derivatives to IN [42]. (CoMFA) was performed to analyze three-dimensional quantitative structure-activity relationship (3D-QSAR) of styrylquinoline derivatives. The stability of 3DQSAR models was proved by the analysis of cross-validated and non-cross-validated methods.

## 4. Antiviral Activity of the Styrylquinoline Integrase Inhibitor Class

All compounds were evaluated for their antiviral activities against HIV-1 replication in either Hela or CEM cells. They were tested for their ability to lower the viral charge in culture supernatants and for their eventual cytoxicity. The results are summarized in Table 10–Table 12.

### 4.1. Influence of the quinoline substitution of the (3’,4’-dihydroxyphenyl)-substituted styrylquinoline scaffold on the antiviral activity

Simple quinoline derivatives such as compound **3b **or **7** were found completely inactive (Table 10). These result markedly contrast with the high antiviral potency of the ketone **14** developed by the Merck Laboratories devoid of any substituent at the C-2 carbon center. Introduction of the (3’,4’-dihydroxyphenyl)ethenyl motive (as in **22**) provided some biological activity, although burdened by cytotoxicity. Several compounds in Table 10 displayed moderate antiviral properties while completely devoid of integrase inhibition potency (See for example compounds **26**, **28**, **29**, **30**), it may thus be concluded that the styrylquinoline scaffold interacted with other targets within HIV-1. For example the diacid **30** was found to inhibit the HIV-1 RT with IC_50_ = 10 μM. Such activity may account for the apparent discrepancy observed.

The observation that an additional carboxyl group at the C-7 position of the quinoline nucleus produced efficient antiviral agent was a crucial breakthrough in the design of styryquinoline class. Indeed, styrylquinoline **35 **gave rise to a strong antiviral effect as measured by β-gal assay, whereas no toxicity on either infected or noninfected cells was observed. By contrast either a nitrile group (**34**) or a carbomethoxy group (**54**) induced strong cytotoxicity masking the antiviral effect. These results confirmed that the 8-hydroxy-quinoline-7-carboxylic acid system is required for both good anti-integrase inhibition and antiviral activity. Since the pKa of the carboxyl group is below 4, it may be assumed that the 7-carboxyl group is ionized under physiological conditions. Accordingly, it appeared that a carboxylate ion at C-7 is required for efficient antiviral activity. Complexation of the magnesium ion within the active site of integrase could tentatively be invoked to explain this result. The favourable influence of an acid group on the activity is evident, however, when the acid is in a position C-5 (compound **32**) this effect is less marked. On the other hand, whatever the position of the acid group the cytotoxicity was deeply reduced.

Compounds **43 **and **46 **(Table 10) bearing at the C-7 position isosters of the carboxyl group were found completely inactive, whereas the acid **42** in which the carboxyl group is bound at C-7 through an ethylenic linker preserved some activity [30]. The α-ketoacid **47** displayed a moderate antiviral activity although devoid of integrase inhibition potency. Finally, the styrylquinoline derivative **53** bearing two similar dihydroxyphenylethenyl subunits was found inactive. This result revealed that contrary to the integrase inhibition structure-activity relationship criteria, *ex vivo* the available space for the substituent at C-7 is limited.

The dichloro and dibromo derivatives **60** and **61** displayed high cytotoxicity masking the antiviral activity. Curiously, the introduction of a large bromine atom at C-5 (**62**) induced a dramatic decline of the antiviral potency with respect to the parent compound **35**. Similarly, styrylquinolines **64** which displayed an unsaturated acid at C-5 was poorly active. These two results seemed indicate that the available space for a substituent at C-5 is relatively narrow. On the other hand, highly active integrase inhibitor **63** (70 nM on the strand transfer), in which the carboxyl residue was directly bound on the quinoline ring, recovered a good antiviral activity but without significant improvement compared to the level of activity of **35** [38]. Derivatives **59d,m-o **which were the only compounds with a significant anti-integrase activity among the 7-keto-styrylquinoline subclass were found either weakly active in cell culture assays (**59m,n,o**) or cytotoxic (as **59d**) [33,35].

**Table 10:** Antiviral activity and cytotoxicity of the (3’,4’-dihydroxyphenyl)styrylquinoline derivatives.

*Compd.*	*Antiviral Activity on CEM cells IC_50_ (* *μM)*	*Cytotoxicity IC_50_ (* *μM)*	*Reference*
**3b**	>100.00	>100.00	14
**7**	>100.00	500.00	19
**14**	5.00^a^	1.25	22
**22**	3.60	8.10	14
**26**	12.00	>100.00	25
**27**	50.00	>100.00	25
**28**	20.00	>100.00	25
**29**	20.00	20-30.00	25
**30**	15.00	>100.00	25
**32**	4.00	200.00	19
**34**	NR	6.20	14
**35**	1.30	120.00	14
**42**	10.00	30.00	30
**43**	>100.00	>100.00	30
**46**	>100.00	>100.00	30
**47**	10.00	50.00	32
**53**	95.00	>100.00	14
**54**	NR	9.20	26
**59d**	NR	12.50	35
**59m **	40.00	80.00	33
**59n **	63.00	>100.00	33
**59o **	35.00	>100.00	33
**60**	NR	17.00	14
**61**	0.80	10.00	19
**62**	40.00	150.00	19
**63**	1.60	60.00	34
**64**	35.00	>100.00	34

^a^ MT-4 T-lymphoid cells, NR: not reached.

### 4.2. Modulation of the ancillary ring

While all compounds bearing a simple phenyl or a heteroaromatic ring (**77a–77d**) have proved to be good integrase inhibitors, they were found either toxic or inactive in cell cultures assays. As their unsubstituted counterpart, the monosubstituted styrylquinolines **77e–77j** were found inactive, regardless of the nature of the substituent on the ancillary ring (Table 11). As a general rule, only compounds bearing a hydroxyl group at the *para *position and an other oxygenated function at C-3’ possessed a promising activity. This result confirmed that structural requirement for *ex vivo* activity are much more stringent that for *in vitro* integrase inhibition. The higher the number of hydroxyl groups on the ancillary ring, the better the antiviral activity. However, the presence of three hydroxyl groups (**77t**) induced some cytotoxicity. In this respect, styrylquinoline **77u** constituted the best compromise. 

**Table 11:** Influence of the ancillary ring substitution on antiviral activity.

Compd.	Antiviral Activity on CEM cells IC_50_ (μM)	Cytotoxicity IC_50_ (μM)	Reference
**77e**	95.0	100.0	26
**77f**	50.0	100.0	26
**77g**	NR	43.0	26
**77h**	NR	47.0	26
**77i**	110.0	>100.0	25
**77j**	57.0	63.0	30
**35**	1.3	>100.0	14
**77l**	>100.0	>100.0	26
**77m**	80.0	>100.0	14
**77n**	30.0	110.0	19
**77o**	30.0	50.0	23
**77p**	10.0	50.0	23
**77q**	3.0	95.0	26
**77r**	54.0	95.0	26
**77s**	62.0	>100.0	14
**77t**	1.2	10.0	14
**77u**	1.5	250.0	26
**77w**	9.0	100.0	26
**77x**	36.0	22.0	37
**77y**	50.0	>100.0	26
**77aa**	6.0^a^	>100.0	19
**77ab**	14.5^a^	>100.0	19
**77ac**	26.0^a^	230.0	19

NR: not reached, ^a^ Hela P4 cells.

### 4.3. Modulation of the spacer

The antiviral evaluation of the linker modified compounds is depicted in Table 12. The reduction of the central double bond which did not affect the integrase inhibition potency dramatically reduced the antiviral activity [26]. Likewise, replacement of the double bond with a simple amide (**92**), or urea (**94,95**) drastically reduced activity. In this context, the amide **93** is a noteworthy exception [18]. Modulation of the hydroxyl substituent pattern on the ancillary ring did not modify the picture. Only, **96**, **101** and to a lesser extent **98** possessed an antiviral activity. 

**Table 12:** Antiviral activity and cytotoxicity of spacer modified inhibitors.

Compd.	Antiviral Activity on CEM cells IC_50_ (μM)	Cytotoxicity IC_50_ (μM)	Reference
**35**	1.3	>100	14
**78**	NR	61	26
**92**	30.0	>100	18
**93**	2.0	>100	18
**94**	>100.0	70	18
**95**	55.0	>100	18
**96**	2.0	>100	18
**97**	40.0	>100	18
**98**	10.0	>100	18
**99**	>100.0	>100	18
**100**	25.0	>100	18
**101**	4.0	>100	18
**102**	>100.0	>100	18
**103**	45.0	>100	18	
**104**	>100.0	>100	18	
**105**	>100.0	>100	18	
**106**	>100.0^a^	>100	19

NR: not reached, ^a^ Hela P4.

## 5. Conclusions

DNA binding assays and computational docking data have highlighted a competitive mechanism between SQLs and the viral DNA end, which leads to the inhibition of the 3’-processing and makes SQLs representative compounds of the INBI group. In cells, the stable IN-DNA interaction within the preintegration complex (PIC) might preclude inhibitors from acting as competitor of viral DNA from being very effective in the cellular context. Together with the dramatic effectiveness of INSTI compounds, this consideration questions the relevance of further developing INBIs. 

Yet, the following observations temper this conclusion. First, although most 3'-processing inhibitors actually lack antiviral effect, this is not the case for SQLs, which show a moderate activity *ex vivo* [44,50,51]. Second, investigation of the mechanism of viral inhibition by compound **77u** in cells revealed the possibility that the inability of integrase to bind to the viral DNA impairs the formation of the IN/DNA nucleoprotein complex. Finally, the lead compound **77u **proved also to be active against viruses resistant to AZT, lamivudine (3TC), nevirapine, efavirenz and against INSTIs-resistant integrase [50]. In vit*ro* selection of resistant virus identified the two mutants C280Y and (V165I V249I) which differ from those conferring resistance to Raltegravir (N155H, G140S, Q148H, Y143) [52,53]. It is not clear how these mutations promote resistance to SQLs. V165 has been proposed to participate in the nuclear translocation of the integrase [54], a step that might be impaired in cells by SQL as observed *in vitro *[51]. In agreement with this difference of mechanism of action, SQLs demonstrated an additive to moderate synergic effect with INSTIs [55].

Altogether, these results evidence that INBIs could be used in combination with other antiretroviral drugs. Considering that resistance has already emerged against INSTIs, they justify pursuing the effort to improve SQLs.
